# Ferroptosis and metabolic syndrome and complications: association, mechanism, and translational applications

**DOI:** 10.3389/fendo.2023.1248934

**Published:** 2024-01-08

**Authors:** Dongmei Zhou, Peipei Lu, Xianglai Mo, Bing Yang, Ting Chen, You Yao, Tian Xiong, Lin Yue, Xi Yang

**Affiliations:** ^1^ Department of Endocrinology, Geriatric Endocrinology and Metabolism, Guangxi Key Laboratory of Precision Medicine in Cardio-Cerebrovascular Diseases Control and Prevention, Guangxi Clinical Research Center for Cardio-Cerebrovascular Diseases, First Affiliated Hospital, Guangxi Medical University, Nanning, China; ^2^ School of Nursing, Hunan University of Medicine, Huaihua, China

**Keywords:** ferroptosis, metabolic syndrome, T2DM, atherosclerosis, obesity

## Abstract

Metabolic syndrome is a medical condition characterized by several metabolic disorders in the body. Long-term metabolic disorders raise the risk of cardiovascular disease (CVD) and type 2 diabetes mellitus (T2DM). Therefore, it is essential to actively explore the aetiology of metabolic syndrome (MetS) and its comorbidities to provide effective treatment options. Ferroptosis is a new form of cell death that is characterized by iron overload, lipid peroxide accumulation, and decreased glutathione peroxidase 4(GPX4) activity, and it involves the pathological processes of a variety of diseases. Lipid deposition caused by lipid diseases and iron overload is significant in metabolic syndrome, providing the theoretical conditions for developing ferroptosis. Recent studies have found that the major molecules of ferroptosis are linked to common metabolic syndrome consequences, such as T2DM and atherosclerosis. In this review, we first discussed the mechanics of ferroptosis, the regulatory function of inducers and inhibitors of ferroptosis, and the significance of iron loading in MetS. Next, we summarized the role of ferroptosis in the pathogenesis of MetS, such as obesity, type 2 diabetes, and atherosclerosis. Finally, we discussed relevant ferroptosis-targeted therapies and raised some crucial issues of concern to provide directions for future Mets-related treatments and research.

## 1 Introduction

Metabolic syndrome is a clinical condition characterized by the presence of numerous metabolic disorders in the same individual, including central obesity, insulin resistance (IR), hypertension, and dyslipidaemia. MetS incidence is gradually growing due to the expansion of the social economy, and changes in diet structure, and lifestyle. MetS also increases the risk of CVD (1) and T2DM (2), which is a global social and public concern. Several studies have demonstrated that iron loading (3) and disordered lipid metabolism (4), resulting in lipid deposition and playing an important role in MetS and complications, might help in the design of potential treatment strategies.

Dixon et al. (5) coined the term ferroptosis, which is a novel kind of cell death characterized by an excessive buildup of lipid peroxides due to intracellular iron deposition in 2012. Ferroptosis is distinct from other types of cell death, such as necroptosis, apoptosis, autophagy, and pyroptosis (6, 7) (**Table 1**). Ferroptosis contributes to the pathophysiology of a variety of diseases (8). Numerous metabolic and inflammatory diseases, including obesity (9), T2DM (10), and atherosclerosis (11), have been linked to ferroptosis, which suggests ferroptosis may have therapeutic promise in MetS and sequelae.

**Table 1:** The comparison of ferroptosis, necroptosis, autophagy, apoptosis and Pyroptosis.

Type	Morphological features	Biochemical features	Key genes
Ferroptosis	Cell membrane: plasma membrane complete and blebbing Cytoplasm: mitochondrial volume decreased, cristae decreased or disappeared, membrane ruptured Nucleus: normal nuclear volume and low chromatin cohesion	Intracellular Fe^2+^ level increased, and a large number of ROS was produced, glutathione depletion and glutathione peroxidase 4 activity decreased, lipid peroxide accumulation	GPX4, NRF2, TFR1, FPN1, SLC7A11, P53, ACSL4
Necroptosis	Cell membrane: plasma membrane fracture, spillage of cellular constituent Cytoplasm: widespread swelling of the organelles Nucleus: moderate chromatin condensation,	Drop in ATP levels, activation of RIP1, RIP3, and MLKL release of DAMPs, causing secondary inflammatory responses	RIP1, RIP3, MLKL
Autophagy	Cell membrane: no changes Cytoplasm: double membrane autophagic vacuoles formation Nucleus: no chromatin condensation,	Increased lysosomal activity, degradation of P62 protein, formation of autophagosome, no inflammatory response	ATG5, ATG7, LC3, Beclin-1, DRAM3, TFEB
Apoptosis	Cell membrane: cytoskeletal disintegration, cellular volume reduction Cytoplasm: increase mitochondrial outer membrane permeabilization, formation of apoptotic bodies Nucleus: nuclear pyknosis and fragmentation, chromatin agglutination	Released cytochrome C, activation proapoptotic proteins of the BCL-2 family, forming the apoptosome, initiating the caspase-processing cascade, DNA fragmentation	Caspase, BAX, BAK, Bcl-2, Bcl-XL, MCL-1, Bim, Noxa,BH3, P53,
Pyroptosis	Cell membrane: increased cell size, plasma membrane blebbing and rupture, formation of pore Cytoplasm: formation of pyroptosis bodies Nucleus: moderate chromatin condensation	Caspase-dependent, gasdermin cleavage, triggers inflammation, releases IL-18 and IL-1β	CASP1, CASP4, CASP5, CASP11, Gasdermin family

GPX4 glutathione peroxidase 4, NRF2 nuclear factor erythroid 2-related factor 2, TFR1 transferrin receptor 1, FPN1 ferroportin 1, SLC7A11 solute carrier family 7 member 11, ACSL4 acyl-CoA synthetase long-chain family member 4, RIP1/3 receptor-interacting serine/threonine kinase 1/3, MLKL mixed lineage kinase domain-like protein, ATG5/7 autophagy-related 5/7, DRAM3 damage regulated autophagy modulator 3, TFEB transcription factor EB, Caspase cysteine-aspartic acid proteas, Bcl-2 B cell lymphoma 2, Bcl-XL B-cell lymphoma extra-large, MCL-1 myeloid cell leukaemia 1, Bcl-XL B-cell lymphoma-extra large, BAK BCL-2 antagonist killer, Bim Bcl-2 protein 11, Bim Bcl2-like11, Noxa NADPH oxidase activator; BH3, Bcl-2 homology 3.

In recent years, an increasing number of studies have elucidated the significant involvement of iron metabolism in the aetiology and progression of metabolic syndrome and its associated complications. The presence of iron overload creates an inherent environment conducive to the manifestation of ferroptosis. Multiple investigations have demonstrated the pivotal role of ferroptosis in the investigation of metabolic disorders, encompassing obesity, diabetes, and cardiovascular ailments. Consequently, comprehending the mechanistic underpinnings of ferroptosis assumes paramount importance in the exploration of metabolic syndrome and its ensuing complications. This review provides a comprehensive overview of the recent advancements in understanding the mechanism of ferroptosis and explores the regulatory aspects of ferroptosis in MetS and its associated complications. Additionally, it delves into the identification of potential drug targets associated with ferroptosis in MetS and its complications. Furthermore, it presents a compilation of drugs or small molecules that exhibit the capability of alleviating MetS and its complications by targeting ferroptosis. The primary objective of this review is to offer some inspiration for the treatment of MetS and its complications.

## 2 Mechanism of ferroptosis

### 2.1 Iron metabolism disorder

**Figure 1:**
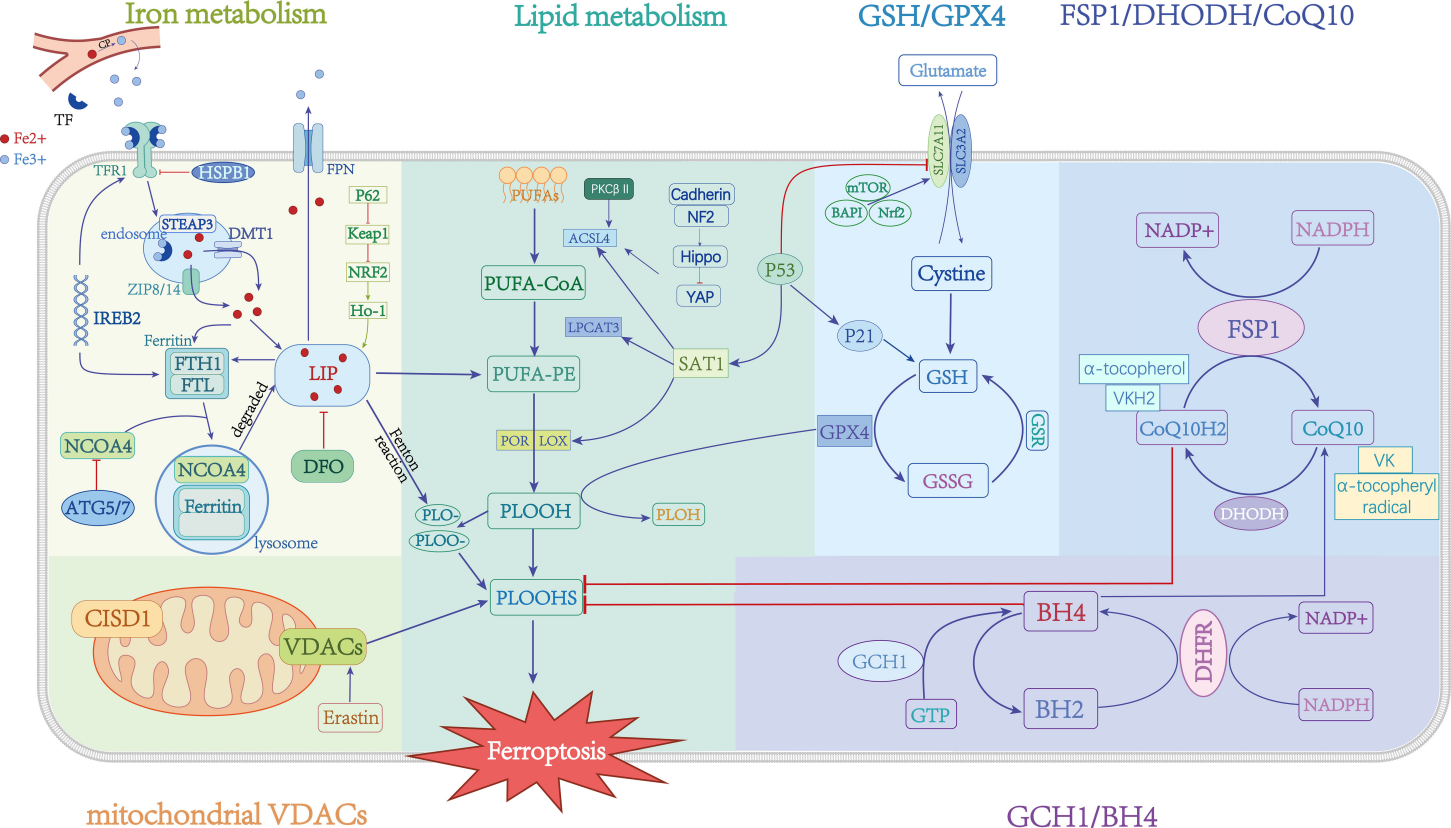
Mechanisms and critical regulators of ferroptosis. →: active; ⟞: inhibit.

Iron is one of the micronutrients needed by the body for metabolism. Iron’s abnormal content and distribution in the body will affect normal physiological processes (**Figure 1**). Iron is mostly contained in the body in the forms of Fe^2+^ and Fe^3+^. Ceruloplasmin (CP) oxidizes Fe^2+^ to Fe^3+^ in blood, which combines with transferrin (TF) on the cell membrane and then bounds to transferrin receptor 1 (TFR1) to transport Fe^3+^ intracellularly through endocytosis (12). STEAP3 converts intracellular Fe^3+^ to Fe^2+^ in the endosome. Zinc iron regulatory protein family 8/14 (ZIP8/14) or divalent metal transporter protein 1 (DMT1) mediates Fe^2+^ release into the cytoplasm. In the end, Fe^2+^ is stored in ferritin or iron pools. Ferritin is made up of two chains: a light chain (FTL) and a heavy chain (FTH). FTH contains an oxidase activity that retains iron in the nontoxic Fe^3+^ form (13). Finally, ferroportin 1 (FPN1) transports Fe^2+^ extracellularly to maintain cellular iron homeostasis, which could prevent oxidative damage. The mechanisms of disordered iron metabolism promote ferroptosis that: under weak acid conditions, the presence of free Fe^2+^ can facilitate the formation of lipid peroxides (PLOOH) by transferring electrons to intracellular oxygen and generating alkoxy radicals through the Fenton reaction (14). Additionally, Fe^2+^ can activate certain iron-dependent enzymes involved in lipid metabolism, such as lipoxygenase (LOXs) (15) and cytochrome P450 oxidoreductase (POR) (16), which catalyze the conversion of phosphatidylethanolamine (PUFA-PEs) into PLOOH, thereby inducing ferroptosis.

Research has demonstrated that the dysregulation of iron-related transporters, including DMT1, FPN1, IRP1, and hepcidin, contributes to the accumulation of labile iron within cells and facilitates the occurrence of ferroptosis (17–20) (**Figure 1**). The phosphorylation of heat shock protein beta-1 (HSPB1) mediated by protein kinase C (PKC) inhibits the iron uptake mediated by transferrin receptor 1 (Tfr1), thereby exerting regulatory control over ferroptosis (21). Furthermore, it suppresses the expression of iron response element binding protein 2 (IREB2), which encodes the important iron metabolism regulators TRFC, FTH1, and FTL, and alleviates ferroptosis sensitivity (5). The intracellular iron content can be modulated by the autophagic breakdown of ferritin, subsequently influencing cellular susceptibility to ferroptosis (22). NCOA4, a nuclear receptor coactivator, facilitates the transportation of ferritin to lysosomes for degradation, resulting in an elevation of cellular Fe^2+^ levels and the induction of ferroptosis. Conversely, the inhibitory effect of ATG5/7 on NCOA4 can impede this process (23). Iron chelation has been demonstrated to inhibit erastin-induced cell death (24). CDGSH iron-sulfur structural domain 1 (CISD1) regulates cellular ferroptosis by mediating mitochondrial lipid peroxidation (25). Moreover, the p62-Keap1-NRF2 pathway can efficiently regulate the generation of intracellular iron ions and ROS, and it also plays a role in ferroptosis regulation (26). These data point to the iron-dependent nature of ferroptosis. The data presented in this study indicate that the regulation of key factors in the intracellular iron metabolism pathway can potentially control the occurrence of ferroptosis.

### 2.2 Lipid metabolism: lipid drivers of ferroptosis via ACSL4 and LPCAT3

One of the main causes of cell ferroptosis is the abnormal accumulation of lipid peroxides. Arachidonic acid (AA) and adrenic acid (ADA) are polyunsaturated fatty acids (PUFAs) found in phospholipids (PLs), which readily react with ROS to cause lipid peroxidation (27) (**Figure 1**). The Acyl-CoA synthetase long-chain family member 4 (ACSL4) preferentially activates PUFAs to form PUFA-CoA derivatives, while LPCAT3 then catalyses the biosynthesis of PUFA-CoA and membrane PEs to phosphatidylethanolamines (PEs), which are finally oxidized to lipid peroxides (PLOOH) by LOXs or PORs (28) (**Figure 1**). Toxic aldehydes such as 4-hydroxy-2-nonenal (4-HNE) or malondialdehyde (MDA) are produced during lipid peroxide catabolism and combined with continuous oxidation reactions on the cell membrane and plasma membrane. The destruction of lipid membranes, which causes cell death, is known as membrane lipid peroxidation.

Recent research has revealed that ACSL4 is a crucial lipid regulator of ferroptosis in cells (29). Dixon et al. (30). conducted an experiment in which they deactivated ACSL4 and LPCAT3 in the haploid cell line KBM7. They discovered that these cells exhibited resistance to two different GPX4 inhibitors, namely RSL3 and ML162. Sebastian et al. (31). conducted a study in which they established ACSL4 knockout (KO) cells and observed a down-regulation in the sensitivity or resistance of these cells to ferroptosis. However, when these ACSL4 KO cells were supplemented with exogenous AA or AdA (along with other long-chain PUFA), their sensitivity to ferroptosis was restored. Additionally, the researchers successfully constructed ACSL4 and GPX4 double knockout cells, which were able to survive and proliferate for extended periods in cell culture. Notably, it is important to highlight that no other cell type has been able to survive in normal culture without GPX4 thus far. In a separate study, Zhang et al (32). demonstrated that protein kinase CβII (PKCβII) plays a crucial role as a sensor of lipid peroxidation. Upon activation, PKCβII triggers phosphorylation and activation of ACSL4 (pACSL4), leading to the inclusion of PUFA into the PL and subsequent cell death. These findings suggest that the PKCβII-ACSL4 pathway contributes to the process of ferroptosis by amplifying lipid peroxidation. Additionally, the findings demonstrate that the Cadherin-NF2-Hippo-YAP pathway plays a role in determining the sensitivity to ferroptosis by modulating the expression of ACSL4 in response to intercellular contact (33). Consequently, ACSL4 may share more similarities with caspase 3, the executor of apoptosis, rather than a housekeeping protein. This suggests that the activation of fatty acids to COA esters is a crucial regulatory step in the process of ferroptosis. In PE biosynthesis and remodeling, LPCAT3 is crucial. The expression of LPCAT3 was downregulated in the cells, further decreasing the lipid peroxide substrates and eventually preventing cell ferroptosis (30). ACSL4 and LPCAT3, which play a central role in PUFA activation and incorporation into PLs, are thought to be lipid drivers of ferroptosis. P53 is a tumor suppressor gene that controls ferroptosis through the p53-SAT1-ALox15 pathway. SAT1, a transcriptional target of P53, is a crucial speed-limiting enzyme for the breakdown of polyamines. SAT1 activation increases the expression of arachidonic acid lipoxygenase 15 (ALOX-15) while also triggering lipid peroxidation and ferroptosis (34). ACSL4 and LPCAT3, which play a central role in PUFA activation and incorporation into PLs, are thought to be lipid drivers of ferroptosis.

### 2.3 A dysregulated ferroptosis defense system causes a buildup of lipid peroxide

#### 2.3.1 GPX4/SLC7A11 pathway inhibits ferroptosis

Glutathione and its associated peroxidase are crucial components of the cellular antioxidant system (**Figure 1**). The cystine/glutamate transporter exchanges glutamate and cystine 1:1 into and out of cells (system Xc-, consisting of SLC3A2 and SLC7A11). In cells, absorbed cystine is converted to cysteine and takes part in the production of glutathione (GSH) (35). The sole enzyme in the body that converts lipid peroxides to alcohols and prevents the buildup of lipid peroxides is called GPX4 (36). As a result, when system Xc- activity is suppressed, GSH depletion or synthesis is hindered, and GPX4 activity is downregulated or degraded, which causes an accumulation of lipid peroxide and triggers ferroptosis (37). The deficiency of selenium, a vitamin employed as a synthetic substrate for GPX4, was discovered to also cause cell ferroptosis (38). Furthermore, p53 directly targets GSH by regulating p21, which increases intracellular GSH and GPX4 production (39). P53 also inhibits relative expressions of system Xc- by downregulating SLC7A11 transcription levels (40). It has been shown that mTOR and BAP1 are both involved in mediating ferroptosis through the regulation of SLC7A11 expression levels (41, 42).

#### 2.3.2 FSP1-CoQ10/DHODH-CoQ10 pathway suppresses ferroptosis

Ferroptosis suppressor protein 1 (FSP1) and CoQ10 inhibit ferroptosis independently of GPX4 (**Figure 1**). Doll et al. (year) conducted a study which revealed that overexpression of FSP1 in cells conferred immunity against ferroptosis. This resistance was observed even in GPX4 KO cells, indicating that FSP1 does not impede the classical ferroptosis mechanism. Subsequent investigations have demonstrated that the anti-ferroptosis function of FSP1 is associated with CoQ10 (43). Specifically, the removal of lipid peroxide intermediates leads to the oxidation of reduced CoQ10, necessitating the reconstruction of its reduced form through the utilization of NADPH. FSP1 employs nicotinamide adenine dinucleotide phosphate (NADPH) to regenerate the reduced form of CoQ10 (44). Interestingly, vitamin K (VK) possesses redox-active properties and shares a structural resemblance to ubiquinone (45). The investigation revealed that FSP1 employs NAD(P)H to catalyze the reduction of VK into vitamin K hydroquinone (VKH2), which effectively sequesters free radicals and impedes lipid peroxidation (46). It is noteworthy that FSP1 employs a comparable mechanism to reduce α-tocopherol radicals, thereby thwarting lipid peroxidation and consequently inhibiting ferroptosis (43). Furthermore, FSP1 exhibits the ability to enhance cellular membrane repair by promoting ESCRT-III, thereby exerting an inhibitory effect on ferroptosis (47). Similarly, Dihydroorotate dehydrogenase (DHODH), which has been identified as a mitochondrial repressor of ferroptosis functions by reducing mitochondrial CoQ10, akin to the role of FSP1 (48).

#### 2.3.3 GCH1-BH4 pathway inhibits ferroptosis

Tetrahydrobiopterin (BH4) is a lipophilic antioxidant known for its ability to protect lipid membranes against autooxidation. Recent research has indicated that BH4 may also have a role in ferroptosis, independent of the GPX4/SLC7A11 pathway (**Figure 1**). Kraft et al (49). conducted a CRISPR activation screening and discovered that GCH1, an enzyme, can catalyze GTP to BH4. GCH1 is considered the rate-limiting enzyme in BH4 synthesis. Subsequent investigations demonstrated that increasing BH4 production through overexpression of GCH1 or exogenous supplementation could effectively rescue cells from ferroptosis. Moreover, studies have shown that the metabolism of GCH1/BH4 was found to inhibit erastin-induced ferroptosis by suppressing NCOA4-mediated ferritin autophagy (50). Furthermore, GCH1 also could remodel lipids and increase the level of reduced CoQ10 while depleting PUFA-PL, which is vulnerable to ferroptosis (51). High quantities of GCH1 or DHODH made cells more resistant to ferroptosis, but low levels made them more susceptible.

#### 2.3.4 Other avenues

Recent research has found additional pathways that suppress ferroptosis. The amino acid oxidase gene (IL4i1) inhibits ferroptosis by scavenging free radicals and coordinating ferroptosis-attenuating gene expression profiles (52). By breaking down peroxide lipids to lessen lipid accumulation, calcium-independent phospholipase (iPLA2β) prevents ROS-induced ferroptosis (53). Interestingly, recent literature has suggested that the sex hormone pathway plays a role in the monitoring mechanism of ferroptosis, which is independent of the GPX4 pathway. Studies have demonstrated that MBOAT2 selectively catalyzes the formation of monounsaturated fatty acids (MUFAs) to PE-MUFA, leading to a reduction in intracellular PE-PUFA and subsequent inhibition of ferroptosis. Additionally, MBOAT1 can also inhibit ferroptosis by catalyzing intracellular phospholipid remodeling. It is worth noting that MBOAT1 and MBOAT2 are regulated differently, with MBOAT1 being regulated by the ER signal and MBOAT2 being regulated by the AR signal. Interestingly, MBOAT1 was highly expressed in female cancers, while MBOAT2 was the opposite (54). These findings provide new ideas for the treatment of ferroptosis.

### 2.4 Other avenues

Voltage-dependent anion channel (VDAC) is crucial in controlling ferroptosis because it transfers ions and metabolites (**Figure 1**). Yagoda et al. (55) found that erastin affects mitochondrial VDACs, altering the membrane permeability and producing a significant release of oxides that ultimately result in cell ferroptosis.

## 3 Inducers and inhibitors of ferroptosis

The currently available ferroptosis inducers are classified into four categories (**Table 2**). Class 1 causes ferroptosis by inhibiting system Xc-, resulting in intracellular GSH depletion and buildup of phospholipid peroxide. Erastin (56) and its analogues Imidazole ketone erastin (IKE) (57), piperazine erastin (PE) (58), sorafenib (59) and sulfasalazine (60) are examples. Class 2 causes ferroptosis by directly inhibiting the enzymatic activity of GPX4. RSL3 causes ferroptosis by reducing the function of GPX4 by covalently binding to its active site (61). Other chloroacetamide containing GPX4 inhibitors can also induce ferroptosis (27). FIN56 promotes GPX4 protein degradation (62). The third group includes lipid peroxidation inducers. FINO2 (63), artemisinin derivatives (64) and artesunate (65) are organic peroxides that promote lipid peroxidation while indirectly inactivating GPX4 to cause ferroptosis via iron oxide. Tert-butyl hydroperoxide (t-BuOOH) is an analogue of lipid peroxide that can stimulate ferroptosis (66). The fourth type is the induction of cellular iron overload to trigger ferroptosis. For example, iron chloride 2, iron citrate, heme, HB and PM2.5 can produce intracellular iron overload and ferroptosis (67–70).

**Table 2:** The common inducers and inhibitors of ferroptosis.

	Mechanisms	Drugs/ molecules
Ferroptosis inducer	(1) Inhibition of system X_C_^–^	Erastin and its analogues (IKE and PE), Sorafenib, Sulfasalazine
	(2) Inhibition of GPX4 enzyme activity	RSL3, FIN56, chloroacetamide-containing GPX4 inhibitors(DPI6, DPI7/ML162, DPI8, DPI9, DPI12, DPI13, DPI15, DPI19),
	(3) Through iron oxide, lipid peroxidation is driven and GPX4 is indirectly inactivated	FINO2, artemisinin and artesunate, t-BuOOH
	(4) Excessive accumulation of non-heme iron (Fe^2+^ and Fe^3+^)	PM2.5, HB, heme, iron chloride 2, iron citrate,
Ferroptosis inhibitor	(1) Inhibit lipid peroxidation	Ferrostatin-1, liproxtatin-1, α-tocopherol
	(2) By chelating iron	DFX, DFP, DFO
	(3) Enzyme inhibitor	Thiazolidinediones, triglyceride C, baicalein, PD146176, Trolox, zileuton
	(4) Inhibition of GPX4 degradation	CDDO, Neurotransmitter dopamine

IKE Imidazole ketone erastin, PE piperazine erastin, RSL3 RAS-selective lethal 3, FIN56 ferroptosis inducing 56, t-BuOOH Tert-butyl hydroperoxide, HB haemoglobin, DFX deferasirox, DFP deferiprone, DFO Deferoxamine, PD146176 15-LOX-1 inhibitors, zileuton ALXO5 inhibitors, CDDO HSP90 inhibitor.

Ferroptosis inhibitors can be divided into four main categories (**Table 2**). The first group acts by inhibiting lipid peroxidation. Ferrostatin-1 (Fer-1) (71), liproxstatin-1 (Lip-1) (72), and α-tocopherol (73) all inhibit ferroptosis. The second class inhibits ferroptosis by chelating iron. Deferasirox (DFX) and deferiprone (DFP) bind to cytosolic iron, and deferoxamine (DFO) promotes the degradation of ferritin into lysosomes (74). The third category is enzyme inhibitors; ACSL4 inhibitors (thiazolidinediones) (31), ALOX inhibitors (baicalein) (75), 15-LOX-1 inhibitors (PD146176) (76), ALXO5 inhibitors (zileuton) (77) and Trolox (78) all suppress ferroptosis. The fourth class inhibits GPX4 degradation. The neurotransmitter dopamine (79) and HSP90 inhibitor (CDDO) (80) prevent GPX4 degradation.

## 4 Ferroptosis and metabolic syndrome and complications

**Figure 2:**
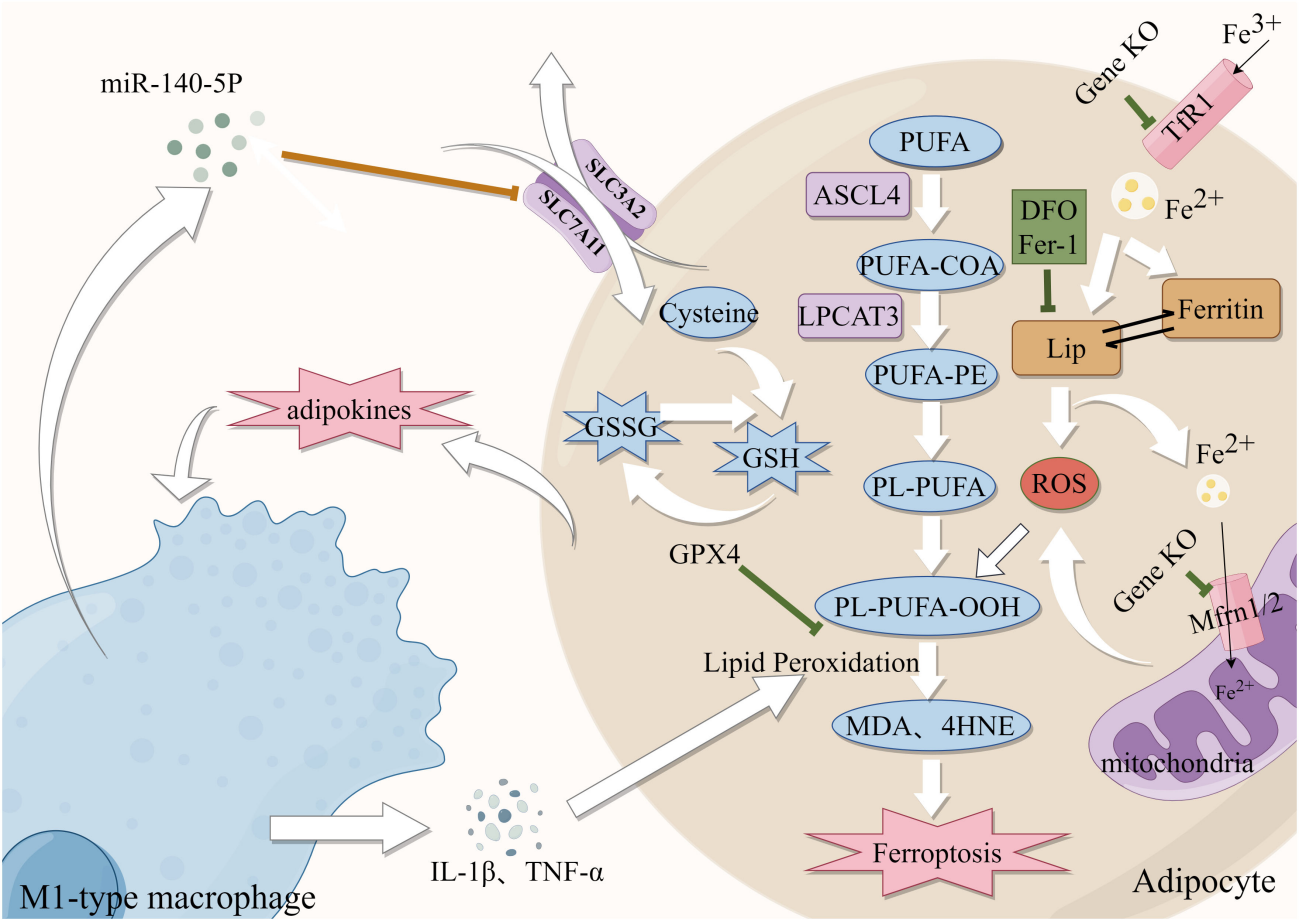
Role of Iron metabolism and ferroptosis in obesity. Green lines: protective factors. →: active; ⟞: inhibit.

MetS is characterized by disorders in glucolipid metabolism and the metabolism of numerous essential components in the body. A large body of evidence suggests that iron overload and disrupted lipid metabolism are major processes in MetS development (**Figure 2**). In theory, these factors supply the inherent underlying conditions for ferroptosis. MetS could increase the risk of T2DM and CVD, especially atherosclerosis. These diseases are caused by the corresponding cells becoming dysfunctional and even experiencing programmed or unprogrammed cell death. CVD and T2DM involve complex pathological processes and multiple cell deaths. So, it is still unclear how iron overload, closely associated with MetS, regulates, or affects what pathways and/or whether it causes iron dysfunction in the corresponding target cells, inducing ferroptosis and thus contributing to disease development.

### 4.1 Ferroptosis and obesity

Abnormal adipose tissue differentiation and accumulation often cause central obesity. A High iron environment may cause aberrant differentiation of adipose tissue, which can lead to metabolic dysfunctions (81). Reduction of iron content through knocked down mitoferritin 1 and 2 (Mfrn1/2) in 3T3-L1 preadipocytes (82), knockout of TfR1 and administration of DFO all resulted in abnormal adipocyte differentiation (83), suggesting that iron availability is required for the development and differentiation of adipocytes. Moreover, animal studies have indicated that lowering iron in white adipose tissue can decrease intestinal lipid absorption and protect mice from the metabolic dysfunction caused by a high-fat diet (HFD) (84). Furthermore, there is additional evidence indicating that the inhibition of adipogenic gene expression and reduction of lipogenesis can be achieved by decreasing intracellular iron levels (85, 86). Moreover, high iron levels contributed to the sustained release of adipokines and promoted the formation of an inflammatory environment, recruited macrophages to infiltrate adipose tissue and induced the development of obesity (87). Obesity increased the polarization of M1-type macrophages in mice, and iron accumulation through the production of inflammatory factors (88). In an interesting finding, miR-140-5p which is found in exosomes released by macrophages that invade adipose tissue decreases GSH production by targeting SLC7A11 to cause ferroptosis (89). The chronic inflammatory state associated with obesity, which is characterized by the excessive release of free fatty acids (FFAs) and proinflammatory cytokines from adipocytes, further supports the notion that ferroptosis is intricately linked to obesity (90). Furthermore, iron chelators reduced adipocyte hypertrophy in polygenic obese mice by interfering with the generation of inflammatory agents and ROS and reducing the infiltration of adipose tissue macrophage (ATM) (91). The above findings support the idea that iron homeostasis plays an important role in the development of obesity through its effects on adipocyte differentiation and function. In conclusion, these findings imply that the manipulation of intracellular iron levels could be a viable approach to addressing obesity.

Obesity is the excessive accumulation and storage of fat in the body, which results in various metabolic problems. Adipose tissue dysfunction is closely linked to iron dysfunction (**Figure 2**). HFD-induced obese mice exhibited higher levels of prostaglandin-endoperoxide synthase 2 (PTGS2) expression and MDA and 4-HNE, as well as lipid ROS and mitochondrial damage (89). ACSL4 and LPCAT3 are lipid drivers that promote the progression of ferroptosis. Elizabeth et al. developed a unique animal model of adipose-specific ACSL4 ablation (Ad-KO), which reduced the levels of PLs, FFAs, and 4-HNE in adipocytes to protect them from increased fat accumulation after feeding HFD (92). It was discovered that in conditionally Lpcat3-deficient mice, there was reduced incorporation of PUFAs (primarily AA) into PLs and redistribution of these FAs to other cellular lipids (e.g., cholesteryl esters) resulted in metabolic disorders and TG accumulation (93). In addition, GPX4 expression was elevated during and necessary for adipocyte differentiation. In several studies, GPX4 overexpression was shown to protect against adipocyte inflammation and hepatopancreatic IR (94). It is noteworthy that Fer-1 treatment mitigated the effects of obesity-induced GPX4 expression (95).Fer-1 reduced iron buildup and lipid peroxidation in HFD-fed mice while increasing SLC7A11 and GPX4 expression (11). These studies suggest that abnormal expression of GPX4, ACSL4 and LPCAT3 in adipose tissue is closely implicated with obesity, and these are also essential regulators of ferroptosis. In conclusion, the preceding results demonstrate that ferroptosis is closely related to obesity, it is suggested that targeting anti-ferroptosis may hold promise as a potential therapeutic strategy for obesity.

### 4.2 Ferroptosis and T2DM

**Figure 3:**
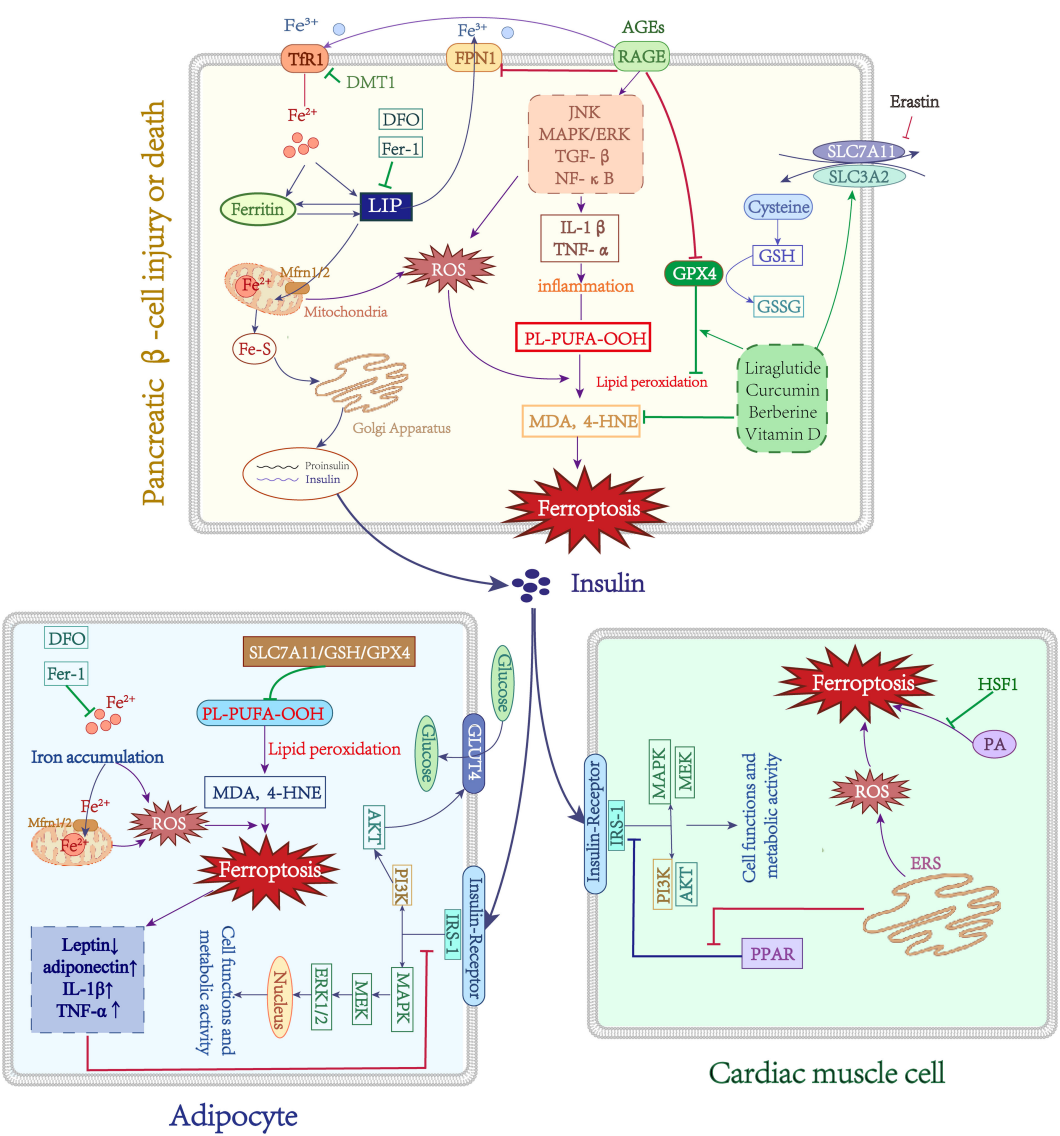
Role of Iron metabolism and ferroptosis in T2DM. Green lines: protective factors. Purple lines: promote ferroptosis or abnormal cell function. →: active; ⟞: inhibit.

The primary pathogenic changes in T2DM are insulin resistance and subsequent progressive secretion defects of pancreatic β-cells, the mechanisms responsible for these are still poorly understood. There is compelling evidence that increases in body iron stores may be associated with T2DM risk (96). This might be linked to impaired insulin secretion, IR, and hepatic gluconeogenesis (97). Insulin resistance (IR) is a complex pathological state characterized by the impaired responsiveness of insulin-dependent cells, such as adipocytes and cardiomyocytes, to normal levels of circulating insulin. This leads to reduced sensitivity of these cells to insulin’s endocrine effects, resulting in diminished uptake and utilization of glucose, the preferred metabolic substrate. The adipocytes secrete various adipocytokines, which regulate whole-body metabolism and homeostasis. Adiponectin and leptin are hormones that are secreted by adipose tissue and regulate insulin sensitivity. Some studies have suggested that under conditions of iron overload, decreased levels of adiponectin (98) and increased secretion of leptin (99) reduce the sensitivity of adipocytes to insulin and lead to insulin resistance (IR). Furthermore, iron-related indicators such as serum ferritin, transferrin, and total iron were evaluated as baseline data in a prospective study, and a positive relationship between IR and baseline serum ferritin levels was discovered after 7 years of follow-up (100). One study demonstrated that DFX selectively stimulated beige fat activation in HFD-induced obese and T2DM mice by upregulating the expression of uncoupling protein 1 (UCP-1), resulting in increased energy expenditure (101). These findings support the notion that elevated iron reserves are linked to adipocyte IR. In addition, there is considerable evidence that iron loading promotes the secretion of inflammatory cytokines by adipocytes (83, 98, 99). TNF-α could prompt IR by increasing the Ser312 phosphorylation of insulin receptor substrate (IRS)-1 (p-Ser312) and decreasing AKT phosphorylation (102). Iron dysfunction leads to mitochondrial dysfunction, which in turn causes IR (103). Moreover, GPX4 knockout animals not only produce adipocyte hypertrophy and macrophage infiltration on their own but also hepatic IR and systemic hypo-inflammation (94). These findings further reveal that iron metabolism problems may disrupt insulin signaling by influencing mitochondrial dysfunction and adipokine production, implying that iron malfunction is implicated in adipocyte IR (**Figure 3**).

In late diabetes, progressive insulin secretion abnormalities emerge, which may be related to islet cell failure. Existing research indicates that islet iron deposition occurs in T2DM patients, which may be associated with the DMT1 increase in cellular iron intake (104). In a T2DM mouse model, abnormal islet morphology, iron deposition in pancreatic tissue, reduced mitochondrial volume, and cristae loss in pancreatic cells were observed, and quercetin partially reversed these damaging effects, implying a role for ferroptosis in pancreatic β-cell dysfunction (105). In the context of type 2 diabetes mellitus (T2DM), iron deposition in the islet tissue has been observed, leading to increased levels of MDA and 4-HNE. This elevation is attributed to the negative modulation of key regulators of ferroptosis, namely SLC7A11, GSH, and GPX4. However, the administration of liraglutide has shown potential in preventing this iron-induced effect (106). Stimulating human islet β-cells with erastin substantially decreased insulin secretion capability; however, pretreatment with Fer-1 or DFO restored islet cell damage (107). Another study found that the multifunctional iron chelator M30 enhanced islet β-cell function in T2DM mice by elevating cerebral hypoxia-inducible factor (HIF-1α) protein levels, promoting insulin signaling and glucose uptake (108). Curcumin can protect mouse MIN6 pancreatic β-cells against the destructive effects of ferroptosis inducers (109). It is known that Berberine improves T2DM and IR through multiple pathways and inhibits ferroptosis by modulating GPX4 in pancreatic cells (110). Moreover, GPX4 overexpression in mice prevents enhanced FFA-induced ROS and lipid peroxidation (111). Vitamin D (VD) has antidiabetic properties, and 1,25D/VDR has been shown that downregulate the expression of FOXO1 to prevent ferroptosis in pancreatic cells (112). Additionally, FA and FAS exhibited protective effects on MIN6 cells against erastin-induced ferroptosis through the upregulation of the Nrf2 antioxidant pathway (113). Finally, ferroptosis may be related to pancreatic cell secretory failure (**Figure 3**). There have been fewer investigations on the function of ferroptosis in pancreatic cell destruction, but ferroptosis inhibition may assist in reducing or preventing diabetes and its consequences.

Cardiomyocytes IR and damage result from the endoplasmic reticulum stress (ERS) interacting with ferroptosis. In a diabetic ischemia/reperfusion (I/R) animal model, observing elevated ERS and ferroptosis markers levels, and Fer-1 therapy reduced ERS, on the other hand, ferroptosis was also downregulated after inhibition of ERS, demonstrating the interplay between ferroptosis and the ERS (114). In addition, ERS caused MIN6 cell ferroptosis and malfunction by suppressing the expression of PPAR and enhancing lipid peroxide accumulation (115). Palmitic acid (PA) induces ferroptosis in cardiomyocytes, and overexpression of heat shock factor 1 (HSF1) prevents this damaging effect by reducing PA-induced lipid peroxidation and regulating the expression of iron metabolism-related genes (116). Moreover, ERS can increase the levels of ROS and trigger oxidative stress, promoting IR. Indeed, oxidative stress and ERS indicate that the cellular oxidative system is dominant, which also provides the intrinsic underlying conditions for ferroptosis, implying that may exert synergistic or interactive effects through a common pathway. However, the exact mechanisms of both still require further investigation.

Persistent hyperglycemia results in the formation of advanced glycation end products (AGEs) through non-enzymatic glycosylation. These AGEs bind to their primary cellular receptor, RAGE, and initiate downstream signaling pathways including JNK, MAPK/ERK, TGF-β, and NF-κB (117–119). This activation leads to the induction of oxidative stress and triggers a cascade of inflammatory responses. On the one hand, the activation of the AGEs/RAGE axis initiates intracellular signaling pathways that result in heightened serine phosphorylation and degradation of IRS-1, consequently obstructing the insulin signaling pathway and leading to insulin resistance (120). On the other hand, the signaling mediated by AGEs/RAGE induces escalated oxidative stress and heightened inflammation within pancreatic β-cells. Reactive oxygen species (ROS) can facilitate the generation and accumulation of hazardous IAPP substances, while RAGE interacts with toxic IAPP intermediates and facilitates the formation of amyloid plaques, ultimately causing toxicity in pancreatic β-cells (121). Intriguingly, recent research has indicated that RAGE contributes to the disruption of iron metabolism and is additionally linked to ferroptosis. Through a bioinformatics analysis of periodontitis and type 2 diabetes, two major signaling pathways (immuno-inflammatory pathway and AGE-RAGE signaling pathway) were identified, with ferroptosis being confirmed as a crucial target for the pathogenesis and treatment of T2DM periodontitis (122). A separate study discovered a negative correlation between the expression of RAGE and the accumulation of lipids and inflammation in the liver. The overexpression of RAGE resulted in a significant increase in the upregulation of Tf/TfR, while the expression of FPN1 decreased, indicating an increase in intracellular iron content. RAGE plays a role in regulating the transport and storage capacity of iron. Additionally, RAGE overexpression led to a decrease in GPX4/GSH, the primary antioxidant pathway in cells, and promoted the production of lipid peroxidation products 4-HNE and MDA. This may result in iron overload, heightened lipid peroxidation products, and the exacerbation of ferroptosis in tissue cells (123). The aforementioned studies provide evidence that RAGE plays a pivotal role in the pathophysiology of diabetic complications, potentially establishing a connection between RAGE, iron homeostasis, and ferroptosis (**Figure 3**). Nevertheless, the existing literature on this subject remains limited, necessitating further investigation into the precise mechanisms involved.

### 4.3 Ferroptosis and atherosclerosis

It is widely recognized that atherosclerosis can result in the narrowing or complete blockage of the arterial lumen, leading to inadequate blood supply, tissue hypoxia, and potential cell death in the affected region. Atherosclerosis is characterized by the presence of lipid-rich plaques and necrotic cells, often accompanied by secondary intraplate hemorrhage, plaque rupture, and local blood clot formation. Extensive clinical and foundational evidence has consistently demonstrated that dyslipidemia, hypertension, obesity, and diabetes are primary risk factors for the development of atherosclerosis. The etiology of atherosclerosis remains uncertain; however, the prevailing theory at present is the endothelial damage-response theory. Hypertension has been implicated in the induction of endothelial damage, while low-density lipoprotein cholesterol (LDL-C) can infiltrate the damaged endothelium and undergo oxidation, resulting in ox-LDL-C. Subsequently, monocytes adhere to and penetrate the intima, transforming into macrophages that engulf ox-LDL-C. These macrophages then transition into foam cells, contributing to the formation of lipid streaks, which ultimately evolve into fibrous plaques.

**Figure 4:**
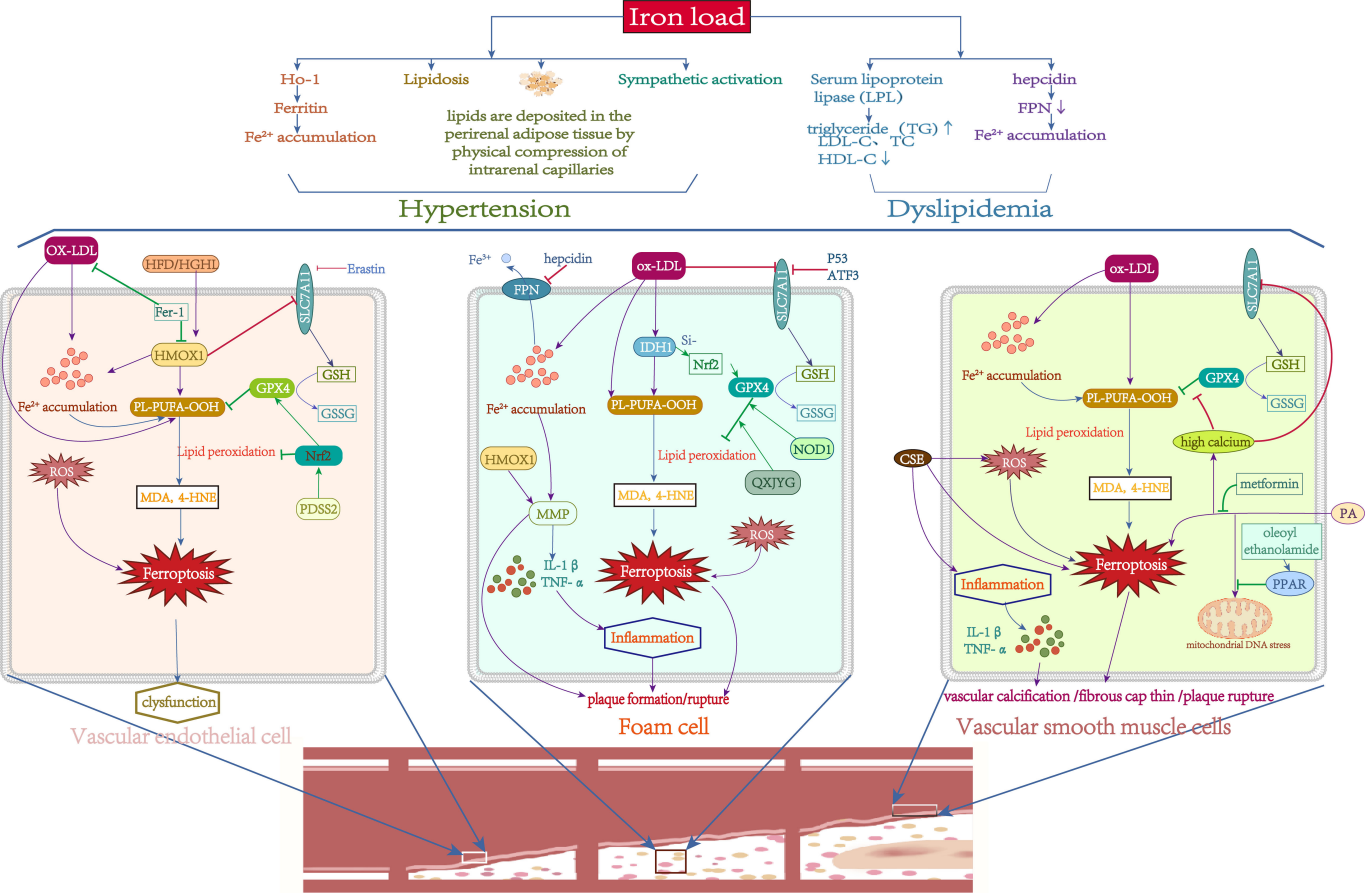
Role of Iron metabolism and ferroptosis in atherosclerosis. Green lines: protective factors. Purple lines: promote ferroptosis or abnormal cell function. →: active; ⟞: inhibit.

Recent research has also highlighted the association between iron overload and abnormalities in lipid metabolism with these established risk factors. Some investigations discovered abnormal iron status in people with obesity/metabolic syndrome-associated hypertension. For example, hemoglobin and transferrin levels were positively associated with blood pressure and hypertension (124). *In vivo*, dietary iron limitation was demonstrated to slow the development of hypertension (125). Increased HO-1 expression promotes haem breakdown and ferritin production, changing intracellular iron distribution. The induction of HO-1 lowers blood pressure in hypertension models, due to HO-1 changing the renal tubular and vascular anatomy, altering renal blood flow (126). As previously stated, iron increases the ectopic deposition of fatty lipids. When lipids are deposited in the retroperitoneal, perirenal, and renal sinus adipose tissue, the effect on intrarenal capillary blood flow through the physical compression of the adipose tissue leads to hypertension (127). When lipids are deposited in vascular tissue, they trigger severe vasoconstriction and oxidative damage, leading to increased blood pressure (128). In addition, SNS activation has an important role in hypertension. Sympathetic activation was found to be enhanced in patients with serum iron overload (129). These findings suggested that iron may also significantly influence the development of hypertension via direct or indirect regulation of adipose tissue, iron metabolism, or the SNS system. Furthermore, research has demonstrated a correlation between dyslipidemia and iron overload. Dyslipidaemia is linked to iron overload. Excess iron consumption worsens hyperlipidemia and can lead to dyslipidaemia in hypertriglyceridaemic/hyperlipidaemic rats (130), mice (131), rabbits (132), and zebrafish (133), notably with a considerable increase in triglyceride (TG) levels. Basic research has found that serum lipoprotein lipase (LPL) activity decreases after iron supplementation, and lowering serum iron in Belgrade rats reduces TG levels (134). Moreover, hepcidin is a hormonal regulator of iron homeostasis. Zhang et al (135). found that the serum hepcidin was elevated in a hyperlipidemia model, which suggests the iron burden in hyperlipidemia may be connected to an unbalanced hepcidin-FPN axis. These findings show an essential relationship between iron metabolism problems, particularly iron overload, and dyslipidaemia (**Figure 4**).

Recently, ferroptosis has been implicated in plaque formation and progression in AS. Studies have found that iron loading can promote AS plaque formation (136). Iron deposition and lipid peroxidation, both of which are hallmarks of ferroptosis, were also identified in advanced atherosclerotic plaques. Inhibition of GPX4 and overexpression of LOXs promote the progression of AS (137, 138). Inhibition of ferroptosis can effectively reduce plaque size and delay the growth of atherosclerotic plaques (139). AS involves complex pathological processes and cell death, including vascular smooth muscle cells, vascular endothelial cells, and macrophages. However, the particular significance of ferroptosis in this setting is uncertain.

The primary cause of AS is endothelial dysfunction. Endothelial cell dysfunction is induced by oxidative damage caused by ROS overproduction. Iron overload was observed to enhance the development of AS in ApoE-/- mice via the ROS pathway (140). Endothelial dysfunction can be caused by oxidized low-density lipoprotein (ox-LDL). In mouse aortic endothelial cells (MAECs), Fer-1 reduced lipid peroxidation and intracellular iron concentrations induced by ox-LDL, whereas SLC7A11 and GPX4 levels increased, suggesting that MAECs can undergo ferroptosis when exposed to ox-LDL (11). The most direct evidence is that erastin can cause vascular endothelial cell (VEC) malfunction to prompt AS via the ROS pathway (141). Additionally, Fer-1 effectively reduced AS in diabetes, which is linked to the haem oxygenase gene (HMOX1) (142). Nrf2 is a key regulator of cellular antioxidant defenses. PDSS2 could drastically reduce ox-LDL-induced ferroptosis in human coronary artery endothelial cells (HCAECs) by increasing Nrf2 expression (143). Taken together, the literature implies that endothelial cell ferroptosis is linked to the pathological process of AS (**Figure 4**), although further research is needed.

Macrophages are the body’s major immune cells and play an essential role in creating and rupturing AS plaques. Macrophages oxidize and phagocytose low-density lipoprotein and accumulate lipid peroxide (LPO) to form foam cells, which promote plaque formation in early AS (144). Iron accumulation in macrophages can accelerate this process (145). More than half of the mostly dead cells in advanced AS are macrophages, which heighten the inflammatory response and vulnerability of the plaque. In severe AS, foam cells eventually experience nonprogrammed or programmed death, including apoptosis, pyroptosis, necrosis, and ferroptosis, which results in plaque and necrotic core development (146). NCF2, NCF, NCF4, MMP9, and ALOX5 were found to be diagnostic indicators of AS in a recent investigation, and these five markers were mostly related to macrophages. Next, these genes were crossed with ferroptosis, pyroptosis and necroptosis gene sets, and the results revealed that NCF2 and ALOX5 were strongly associated with ferroptosis. This was confirmed by basic experiments, indicating that macrophage ferroptosis plays an important role in atherosclerotic plaque core necrosis (147). Macrophage iron excess can enhance MMP production, resulting in extracellular matrix disintegration and plaque rupture, prompting AS development by boosting the inflammatory response (148). Hepcidin increases intracellular iron levels and decreases serum iron levels. A deficit in hepcidin with low intracellular iron levels in macrophages can slow the course of AS (149). P53 and ATF3 may suppress SLC7A11 expression, reducing GSH production and thereby hastening the course of AS (150). Since HMOX1 is highly expressed in AS and associated with MMP production and macrophage infiltration, it may be a diagnostic biomarker for the disease (151). NOD1 plays an important role in immune defense, and overexpression of NOD1 increases GPX4 levels through CXCR4-dependent signaling (152). Isocitrate dehydrogenase 1 (IDH1) is essential for foam cell production. Ox-LDL substantially elevated IDH1 protein levels and promoted ferroptosis and foam cell formation in macrophages, whereas IDH1 inhibition reversed this result by boosting Nrf2 expression (153). Qingxin Jieyu Granules (QXJYG), a comprehensive herbal formulation for the treatment of AS, inhibited ferroptosis via regulation of the GPX4/xCT signaling pathway, thereby slowing AS development and plaque fragility (139). These results can summarize that macrophage ferroptosis plays an important role in early AS plaque development and in late AS plaque rupture (**Figure 4**).

Vascular smooth muscle cells (VSMCs) are the main cell type in atherosclerotic plaques at all stages. In the early stages of AS, VSMCs absorb ox-LDL, resulting in foam cell production, and producing chemokines that attract monocytes, which develop into macrophages. VSMCs create an extracellular matrix to form a fibrous cap in advanced AS. Consequently, VSMC mortality may cause fibrous cap thinning and aggravate plaque instability. Cigarette smoke extract (CSE) can induce ferroptosis and upregulate the expression of inflammatory factors in primary rat VSMCs, suggesting that CSE induces VSMC ferroptosis (154). The most direct evidence is the observation of downregulated GPX4 and upregulated PTGS2 and ACSL4 in human coronary AS tissue (155). In contrast, overexpression of GPX4 in ApoE-/- mice delayed the development of AS by lowering lipid peroxidation (156). In addition, VSMCs also participate in vascular calcification to promote atherosclerotic plaque rupture through many mechanisms. Ye et al (157). discovered that high calcium and phosphate concentrations cause iron mortality in rat VSMCs by blocking the SLC7A11/GSH/GPX4 axis and, hence accelerating vascular calcification. *In vitro*, PA caused VSMC ferroptosis and calcium deposition, while pretreatment with metformin mitigated these effects (158). Additionally, while causing ferroptosis, PA intervention in VSMCs can produce mitochondrial DNA stress, and oleoyl ethanolamide can counteract this harmful impact by activating PPAR, therefore attenuating vascular calcification (159). In conclusion, these findings suggest the active participation of VSMCs in the beginning and progression of AS via ferroptosis caused by several routes (**Figure 4**).

Numerous studies have indeed highlighted the significant involvement of pyroptosis in AS. Pyroptosis is distinguished by the swift disintegration of the plasma membrane, followed by the liberation of cellular contents and proinflammatory mediators, including IL-1β and IL-18 (160). In cardiovascular disease (CVD), the pyroptosis of vascular endothelial cells disrupts the structural integrity of the vessel wall, consequently facilitating lipid deposition and contributing to the development of AS (161). Additionally, pyroptosis of vascular smooth muscle cells results in plaque instability, rupture, and hemorrhage, thereby elevating the susceptibility to acute coronary syndrome and stroke. Furthermore, monocyte/macrophage pyroptosis exacerbates the inflammatory response and fosters the progression of AS. In contrast to the ferroptosis pathway, pyroptosis is initiated by diverse stimuli, such as high fat and high glucose, which induce mitochondrial dysfunction and subsequent reactive oxygen species (ROS) generation. This ROS production triggers the activation of NF-κB, which subsequently activates the NLRP3 inflammasome. Ultimately, the formation of membrane pores occurs, resulting in cell swelling and rupture, leading to the release of cellular contents and mature inflammatory mediators including IL-18 and IL-1β (162). Consequently, pyroptosis contributes to the promotion of inflammation. The diverse mechanisms of cell death suggest distinct therapeutic targets that hold potential for intervention. The demise of cells may be attributed to the combined impact of various programmed cell death mechanisms, such as necroptosis, pyroptosis, and ferroptosis, thereby influencing diverse pathways and biological functions. Consequently, this observation may offer novel perspectives for the concurrent management of diseases. Subsequently, it becomes evident that this synergy exhibits unforeseen intricacy, thus necessitating a comprehensive exploration of diverse pathways and further investigation into the functional mechanisms and applications thereof.

## 5 Ferroptosis: a novel therapeutic strategy

Taken together, a substantial amount of data shows the link between ferroptosis and the development and progression of the aforementioned disorders. Iron loading and lipid peroxide buildup are the major processes controlling susceptibility to ferroptosis among the molecular mechanisms of ferroptosis. Thus, addressing the main features of ferroptosis, namely, iron loading and lipid peroxidation, may be a viable therapeutic strategy for these disorders (**Table 3**). The iron chelators commonly utilized include deferoxamine (DFO), Deferasirox (DFX), and deferiprone (DFP). DFP and DFX function by binding to intracellular iron, whereas DFO operates by facilitating the degradation of ferritin within lysosomes. However, both DFP and DFX can be eliminated through urine or bile. DFO exhibits limited bioavailability and forms a 1:1 ratio with iron, often necessitating subcutaneous or intravenous administration. Conversely, DFP and DFX form complexes with iron in ratios of 3:1 and 2:1, respectively, enabling oral consumption. Phospholipid peroxidation is also a key mechanism for modulating ferroptosis susceptibility; thus, medicines that limit the effects of phospholipid peroxidation (lipophilic antioxidants) might be used as a possible ferroptosis treatment. Fer-1, a specific ferroptosis inhibitor, works by removing ferrous iron from lipid hydroperoxides (71). Lip-1 and α-tocopherol both have anti-ferroptosis actions by preventing the proliferation of lipid peroxyl radicals or destroying the proliferation of the peroxide chain, thereby decreasing disease development (163). Ferulic acid (FA) is a plant-based phenolic acid with low cytotoxicity and high bioavailability that is often found in fruits, vegetables, and certain drinks. It functions as an antioxidant and anti-inflammatory by quenching free radicals and increasing Nrf2 nuclear translocation through its phenolic hydroxyl groups (113). These drugs have shown remarkable efficacy in multiple disease-related ferroptosis models, providing promising treatment strategies for MetS and its complications.

**Table 3:** The potential drugs and mechanisms of ferroptosis-targeted.

Reagents	Key mechanisms	Model	References
Fer-1	Inhibit the iron accumulation, and lipid peroxidation and increase the expressions of SLC7A11 and GPX4	ApoE mice	(11)
Rapamycin	Inhibited cardiomyocyte death, significantly lower ROS production	myocardial infarction	(68)
DFO	Inhibit lipogenesis and reduce the expression of genes related to mitochondrial biogenesis	human and 3T3-L1 adipocyte	(82)
DFO	Reduce reactive oxygen species and inflammatory factors secretion, increase the levels of HIF-1α and antioxidant enzymes	ob/ob mice	(85)
DFO	Suppression of oxidative stress, inflammatory cytokines, and macrophage infiltration	polygenic obese mice	(91)
Fer-1	Attenuate inflammatory cell infiltration, and inflammatory cytokine expression, increase GPX4 expression	obesity	(95)
DFX	Increase the expression of UCP-1, preferentially activating beige fat, increase energy expenditure	obesity and T2DM	(101)
Quercetin	Partially reversed the damage of iron deposition on islet β cells	T2DM mouse	(105)
Liraglutide	upregulate SOD, GSH-PX, and GSH activity and downregulate MDA, 4-HNE, and NOX4 expression	T2DM	(106)
Fer-1/DFO	Ameliorate islet function	islet transplantation	(107)
M30	Increase HIF-1α protein level, upregulation of insulin signalling and glucose uptake, improve the function of pancreatic β cells	T2DM mouse	(108)
Curcumin and EGCG	Prevent GSH depletion, GPX4 inactivation, and lipid peroxidation	Murine MIN6 Pancreatic Beta-Cells	(109)
Berberine	accelerate cell viability and proliferative abilities, reduce the content of Fe^2+^ and ROS	islet β cell	(110)
Vitamin D	1,25D/VDR inhibited pancreatic β cell ferroptosis in T2DM by downregulating the expression of FOXO1	T2DM rat	(112)
FA	Electrons are supplied by the phenol hydroxyl group to quench free radicals and enhance the nuclear translocation of Nrf2	murine MIN6 cells	(113)
Fer-1	reduce ERS and myocardial injury	DIR	(114)
QXJYG	suppress the MDA, enhanced SOD and GSH, and reduced inflammatory factors	ApoE mice	(139)
Fer-1	Ameliorate lipid peroxidation and downregulate ROS production	Diabetic atherosclerosis	(142)
Metformin	Reduced PA induced ferroptosis and calcium deposition in VSMC	Vascular smooth muscle cells	(158)
Oleoyl ethanolamide	Activation of PPAR to counteract PA-induced mitochondrial DNA stress	Vascular calcification model	(159)

ROS reactive oxygen species, HIF-1α Hypoxia induicible factor-1 alpha, SOD superoxide epigallocatechin-3-gallate, QXJYG Qing-Xin-Jie-Yu Granule, UCP-1 uncoupling protein-1, FA ferulic acid.

### 5.1 Obesity

Recent research has demonstrated that deferoxamine ameliorates obesity in animal models through its ability to diminish iron content in adipocytes, impede the production of inflammatory factors, and mitigate macrophage infiltration, consequently influencing adipocyte differentiation (83, 86). In HFD-fed mice, Fer-1 has been observed to mitigate oxidative stress and lipid peroxidation while augmenting the expression of the SLC7A11/GPX4 pathway (11). Liraglutide is a medication that treats obesity and diabetes by acting on the glucagon-like peptide-1 (GLP-1) receptor. Studies have shown that liraglutide regulates Nrf2/HO-1/GPX4 to prevent hepatic iron mortality in db/db mice (106). In summary, the regulation of iron levels and GPX4 may represent potential therapeutic targets for managing obesity in clinical settings.

### 5.2 T2DM

Studies have shown that DFO and Fer-1 can restore islet cell damage (107). Quercetin, a flavonoid, has been identified as a natural modulator of iron metabolism. Research has demonstrated that the administration of quercetin can effectively impede iron deposition and significantly restore the content of GDH and the activity of SOD in pancreatic β-cells (105). Similarly, curcumin, berberine, and vitamin D possess antioxidative properties that can ameliorate T2DM and IR, as well as prevent ferroptosis in pancreatic β-cells through various mechanisms (109, 110, 112). These findings suggest that quercetin, curcumin, berberine, and vitamin D hold promise for potential therapeutic benefits in the context of T2DM. Nevertheless, further clinical trials are imperative to ascertain the safety and efficacy profiles of these pharmaceutical agents.

### 5.3 Atherosclerosis

Hypertension, dyslipidemia, diabetes, and obesity have been identified as risk factors for cardiovascular disease. Research has demonstrated that iron overload can contribute to the onset and progression of these risk factors. Consequently, the regulation of iron levels through dietary interventions and pharmacological agents has emerged as a potential therapeutic approach for cardiovascular disease. Three frequently employed iron chelators are commonly utilized in the management of iron overload cardiomyopathy (164). Deferiprone an orally active iron chelator sanctioned by the FDA, specifically acts upon hemorrhagic iron during ischemia/reperfusion events and assumes a cardioprotective function in cases of acute myocardial infarction by eliminating intramyocardial hemorrhage and diminishing myocardial hypertrophy (165). In the context of doxorubicin-induced cardiomyopathy, Fer-1 and dexrazoxane (DXZ) exhibit notable efficacy in mitigating cardiac injury through the preservation of mitochondrial function (166). Furthermore, it was observed that Fer-1 exhibited a reduction in ox-LDL-induced lipid peroxidation in mouse aortic endothelial cells (MAECs) (11). The mTOR pathway, known for its ability to regulate iron metabolism, has been found to safeguard cardiomyocytes against iron overload by targeting various iron transporters (68). Studies have reported that statins can mitigate ferroptosis in animal models of myocardial ischemia-reperfusion and heart failure (167). Additionally, pretreatment with metformin has been shown to attenuate ferroptosis and calcium deposition in vascular smooth muscle cells (158). These findings indicate that ferroptosis assumes a pivotal role in the pathogenesis of cardiovascular disease, and the modulation of iron levels or the utilization of ferroptosis inhibitor-1 represents a promising avenue for the management of CVD.

## 6 Summary and outlook

Since the concept of ferroptosis was first introduced in 2012, research on ferroptosis has increased tremendously over the last decade, and its mechanisms of occurrence and defense have become increasingly obvious. Ferroptosis is implicated in the pathogenic processes of many illnesses, and medicines that target ferroptosis have been shown to have the desired effects. In this paper, we first reviewed the mechanisms of ferroptosis and the main regulatory mechanisms of ferroptosis inducers and inhibitors. Then, we discussed the relevance of ferroptosis to the development of MetS and its complications, such as obesity, T2DM, and atherosclerosis. Finally, we analyzed the relevant ferroptosis-targeted therapies, which will provide directions for future MetS-related treatment and research.

Although several studies have demonstrated that ferroptosis is intimately linked to the onset and progression of MetS and complications, many questions remain unanswered. First, the inflammatory response, oxidative stress, and ERS play important roles in the pathogenesis of MetS, it’s unclear whether ferroptosis shares common pathways or key regulators with these mechanisms, which could provide new directions for combining different therapeutic interventions. Second, in the MetS setting, various small-molecule drugs, such as iron chelators, ferroptosis inhibitors, and lipid peroxidation inhibitors, have demonstrated promising therapeutic activity. However, numerous preclinical and clinical trials are still required to determine their safety and efficacy. Finally, precisely targeted ferroptosis inhibition is needed, such as systemic iron reduction and overexpression of critical components such as GPX4, which may cause damage to other organs. As a result, identifying these pathways and techniques may be crucial for treating ferroptosis. A scientific basis for targeted treatment of ferroptosis associated with these diseases will be established by addressing these crucial scientific concerns. It will provide important insights into how ferroptosis is involved in these diseases.

## Author contributions

DZ, PL, and XM contributed equally to reviewing the literature and drafting the manuscript. BY, TC, YY, and TX assisted in the literature review and drafting of the manuscript. LY and XY provided critical editing and revisions of the manuscript. All authors contributed to the article and approved the submitted version.
